# Asynchronous pulse responses of soil carbon and nitrogen mineralization to rewetting events at a short-term: Regulation by microbes

**DOI:** 10.1038/s41598-017-07744-1

**Published:** 2017-08-08

**Authors:** Xiaoli Song, Jianxing Zhu, Nianpeng He, Jianhui Huang, Jing Tian, Xiang Zhao, Yuan Liu, Changhui Wang

**Affiliations:** 1 Key Laboratory of Ecosystem Network Observation and Modeling, Institute of Geographic Sciences and Natural Resources Research, Chinese Academy of Sciences, Beijing, 100101 China; 2 State Key Laboratory of Vegetation and Environmental Change, Institute of Botany, the Chinese Academy of Sciences, Beijing, 100093 China; 3 College of Animal Science and Veterinary Medicine, Shanxi Agricultural University, Taigu, 030801 China

## Abstract

Rewetting after precipitation events plays an important role in regulating soil carbon (C) and nitrogen (N) turnover processes in arid and semiarid ecosystems. Here, we conducted a 48-h rewetting simulation experiment with measurements of soil C and N mineralization rates (*R*_C_ and *R*_N_, respectively) and microbial biomass N (MBN) at high temporal resolution to explore the pulse responses of *R*_*C*_ and *R*_N_. *R*_C_ and *R*_N_ responded strongly and rapidly to rewetting over the short term. The maximum *R*_C_ value (because of pulse effects) ranged from 16.53 to 19.33 µg C g_soil_^−1^ h^−1^, observed 10 min after rewetting. The maximum *R*_N_ varied from 22.86 to 40.87 µg N g_soil_^−1^ h^−1^, appearing 5–6 h after rewetting. The responses of soil microbial growth to rewetting were rapid, and the maximum MBN was observed 2–3 h after rewetting. Unexpectedly, there was no correlation between *R*_C_, *R*_N_, and MBN during the process of rewetting, and *R*_C_ and *R*_N_ were uncoupled. In sum, the pulse responses of *R*_C_, *R*_N_, and microbial growth to simulated rewetting were rapid, strong, and asynchronous, which offers insights into the different responses of microbes to rewetting and mechanisms behind microbes.

## Introduction

Periodic rewetting of soil after precipitation events is common and exerts a significant influence on the carbon (C) and nitrogen (N) cycles of an ecosystem^1–3^. Precipitation events are the main drivers of the N cycle in soil and plant growth in arid and semiarid areas, which all depend on precipitation intensity and previous soil water conditions^2–4^. Birch H.F^5^ first reported that precipitation events after long-term or severe drought lead to strong and rapid increases in soil respiration and microbial biomass through a pulse or priming effect. Huxman, *et al*.^1^ reviewed the pulse effects of precipitation events on plants, soil, and physiological ecosystem activity and found significant impacts on the C and N cycles in soil and productivity in arid and semiarid ecosystems where small precipitation events can trigger these pulse effects. Borken and Matzner^6^ reappraised the effects drying-wetting effects on soil C and N mineralization and fluxes, and found that increasing summer droughts may reduce the mineralization and fluxes of C and N whereas increasing summer precipitation can enhance the losses of C and N from soils. Global climate models predicted that the frequency and intensity of drying-rewetting events are likely to alter due to future climatic change^7^. These future scenarios highlight the importance of understanding how drying-rewetting cycles will influence dynamic of soil C and N cycles^7, 8^. For example, a recent meta-analysis study that investigated drought effects on soil C cycles and found that greater C were loss from C-rich (>2% organic C) soil during whole drying-rewetting cycles^8^.

Previous studies have explored different aspects of pulse effects in arid and semiarid areas^9, 10^. Birch^9^ found that those soils subjected to short-term drought may experience greater C and N mineralization rates after a short period of rewetting than these soils with constant soil water content. Sponseller^10^ investigated the rapid pulse responses of microbes and soil respiration to short-term precipitation in the Sonoran Desert at intervals of 15 and 30 min and 1, 2, 5, 24, and 48 h. Dijkstra *et al*.^11^ found that microbial fixation, total soil N mineralization, and nitrification rate increased rapidly within one to three days. As proposed by Huxman, *et al*.^1^, the pulse effects of precipitation events are time-sensitive and may be classified as short-term sharp rise (minute or hour) or long-term stable increase (day or month). Jones and Murphy^12^ found that the response of soil microbes to added artificial substrates (sugars and amino acids) and rainwater separately was instantaneous, peaking in less than 16 min, which suggested that the rapid responses of soil C and N mineralization to rewetting are rapid and strong at initial stage. To date, however, no study has focused on the short-term pulse effects of precipitation events or rewetting on both soil C and N turnover from minutes to several days.

Soil C and N mineralization, driven by microbial activity, are often closely linked without the limitations of soil water content^13–15^. In the process of rewetting, microorganisms play two important roles as regulators of soil C and N mineralization and reservoirs of soil nutrients^1, 16^. Through the pulse effects of rewetting, microbial biomass and activity in soil first increase sharply, rapidly decomposing soil organic matter (SOM) to satisfy microbial requirements for energy and elements (C, N, and P) within a very short time. The disproportional C:N ratios of SOM and soil microbes result in a sharp increase in soil CO_2_ emissions due to the relative stability in C:N ratios required by soil microbes^17, 18^. After rapid microbial growth, soil N mineralization rates are expected to increase as a result of an increase microbe metabolization and microbe death accompanying with the depletion of labile SOM^5^. Finally, the C:N ratios arrive at the relatively stable status required by microbes, and the often coupled ratios of soil C and N mineralization may be observed, as in previous studies^13, 19^. We therefore assumed that the different roles of soil microbes under different phase of rewetting may result in uncoupled C and N mineralization at a short-term. Unfortunately, few studies have explored the coupled relationship between soil C and N mineralization during the initial phase of rewetting event^1, 9–11^.

Based on a 48-h incubation experiment of soil C mineralization rate (*R*_C_, 6-min intervals), N mineralization rate (*R*_N_, 18 times), and microbial biomass nitrogen (MBN, 18 times) at high temporal resolution, we investigated the dynamics of *R*_C_, *R*_N_, and MBN in two semi-arid forest soils through a simulation of rewetting. The main objectives of this study were (1) to investigate the short-term dynamics of *R*_C_, *R*_N_, and MBN after rewetting; (2) to quantify the pulse effects of simulated rewetting on *R*_C_ and *R*_N_; and (3) to discuss the underlying mechanisms influencing *R*_C_ and *R*_N_ in initial phase of rewetting event. We specifically tested the hypothesis that the short-term responses of soil microbes to rewetting may drive different processes of and an uncoupled relationship between *R*_C_ and *R*_N_.

## Results

### Dynamics of *R*_C_ and pulse effects of rewetting

**Figure 1:**
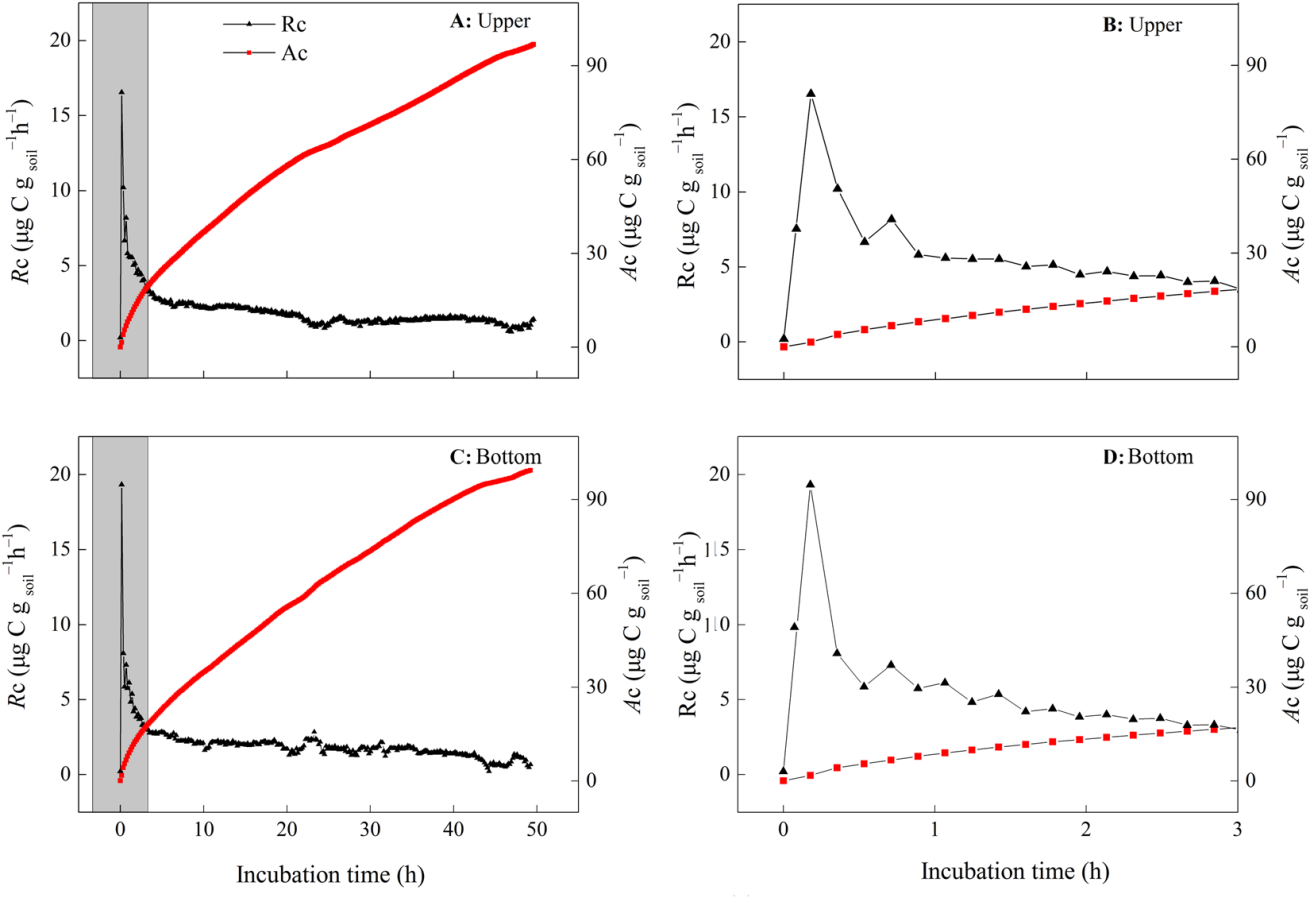
Dynamics of soil carbon mineralization rates (*R*_C_, μg C g_soil_^−1^ h^−1^) and accumulation (*A*_C_, μg C g_soil_^−1^) through simulated rewetting. Panels B and D were the amplification of shades in panels A and C, respectively. Upper and Bottom represented the sampling locations along a slope.

**Figure 2:**
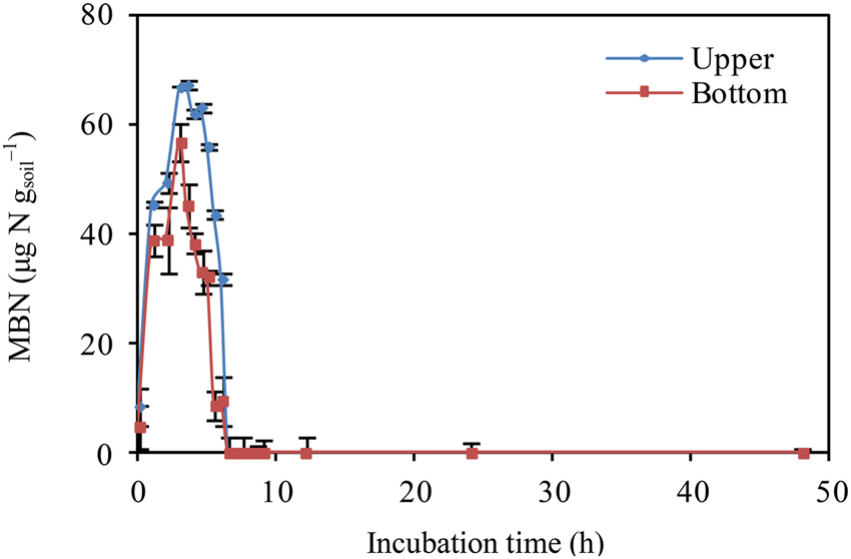
Dynamics of soil microbial biomass nitrogen (MBN, μg N g_soil_^−1^) after rewetting.

*R*_C_ increased immediately and sharply, peaking after 10–20 min of simulated rewetting (Fig. 1), and then apparently decreased with prolonged incubation, irrespective of location (upper or bottom). Similar trends were observed with MBN, although a time lag was apparent, with a peak about 3–4 h after rewetting (Fig. 2). No significant relationships between *R*_C_ and MBN were observed from the early stages through the peaking of *R*_*C*_ during the 48-h incubation period.

**Figure 3:**
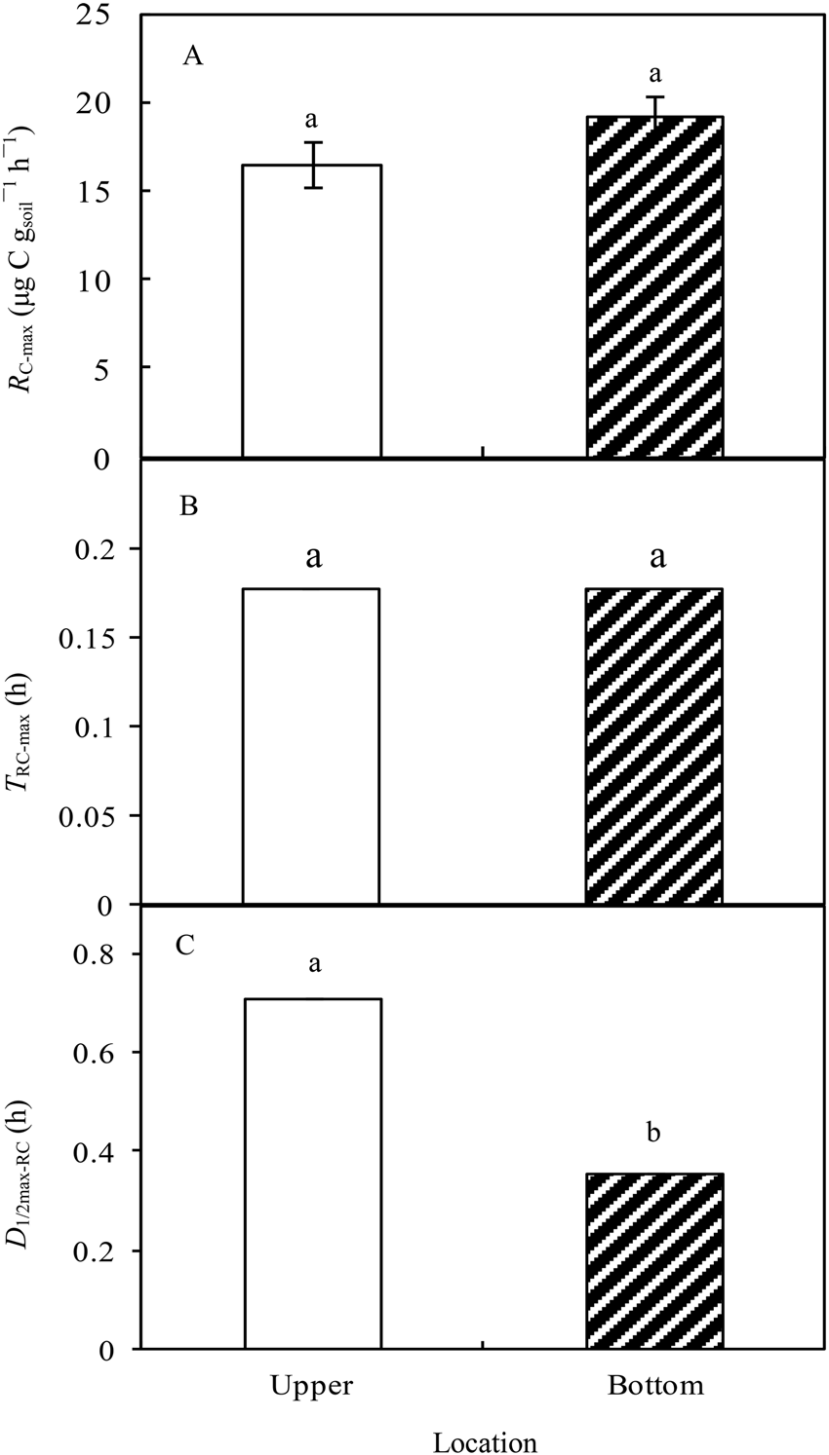
Pulse responses of soil carbon mineralization to simulated rewetting: (**A**) maximum soil carbon mineralization rate (*R*_C-max_, μg C g_soil_^−1^ h^−1^), (**B**) time to reach *R*_C-max_ (*T*_RC-max_, h), (**C**) duration from 1/2 *R*_C-max_ to 1/2 *R*_C-max_ (*D*_1/2max-RC_, h) being used to express the duration of pulse effect. Data with same superscript letters within each column indicate no significant difference at *P* = 0.05.

Rewetting exerted strong pulse effects on *R*_C._ The values of *R*_C-max_ were 16.53 and 19.33 μg C g_soil_^−1^ h^−1^ for the upper and bottom samples, respectively, with no significant difference between the locations (Fig. 3A). *T*_RC-max_ was observed at 0.18 h in both locations (Fig. 3B). *D*_1/2max-RC_ was significantly higher in the upper (0.71 h) than the bottom location (0.36 h) (*P* < 0.05, Fig. 3C). *A*_C_ was 109.34 µg C g_soil_^−1^ in the upper location and 114.35 µg C g_soil_^−1^ in the bottom location, showing no significant difference between them (Figures not shown).

### Dynamics of *R*_N_ and pulse effects of rewetting

**Figure 4:**
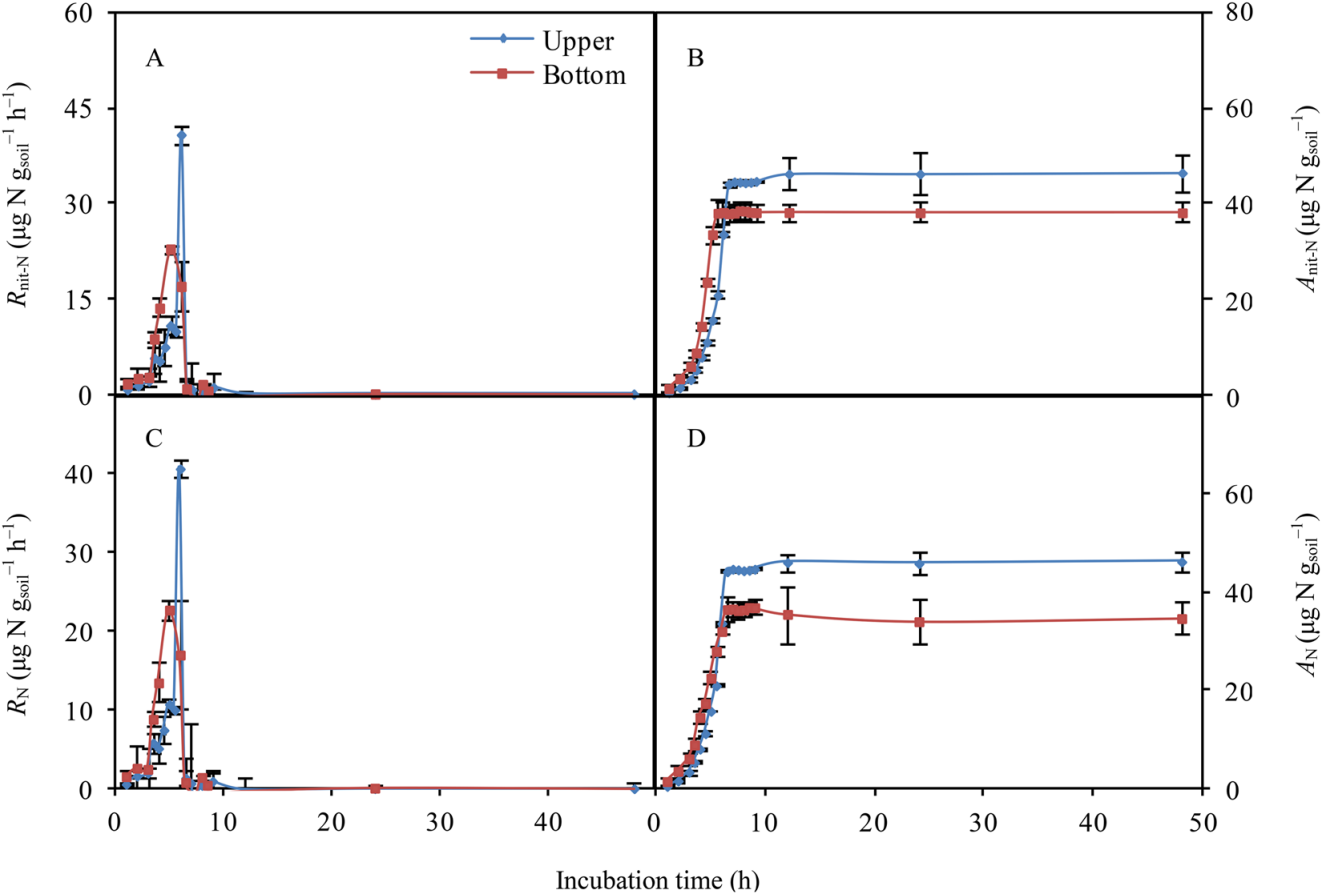
Responses of soil nitrogen mineralization and nitrification to rewetting, (**A**) soil nitrogen nitrification rate (*R*_nit-N_, μg N g_soil_^−1^ h^−1^), (**B**) accumulation of *R*_nit-N_ (*A*_nit-N_, μg N g_soil_^−1^), (**C**) soil nitrogen mineralization rate (*R*_N_, μg N g_soil_^−1^ h^−1^), (**D**) accumulation of *R*_N_ (*A*_N_, μg N g_soil_^−1^).

After rewetting, *R*_N_ first exhibited a stable increase, then a sharp increase, and finally a steep decrease in both the upper and bottom locations (Fig. 4). The results indicated that *R*_nit-N_ was the main contributor to *R*_N_, while the contribution of *R*_amm_-N was small (0.01–0.65 µg N g_soil_^−1^ h^−1^) and we therefore did not show it in Fig. 4.

**Figure 5:**
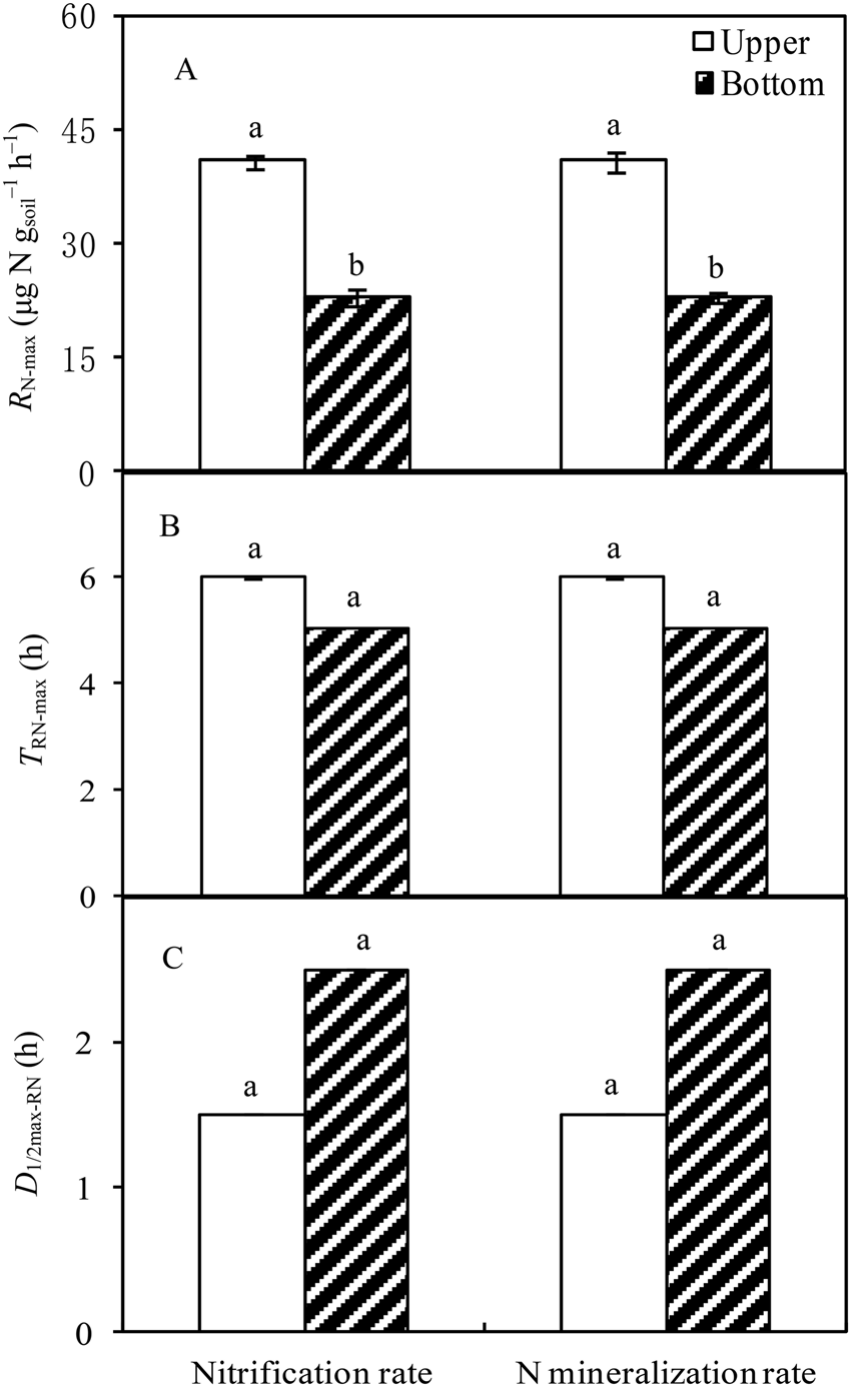
Pulse effects of soil nitrogen mineralization and nitrification from simulated rewetting event, (**A**) maximum soil nitrogen mineralization or nitrification rate (*R*_*N-*max_, μg N g_soil_^−1^ h^−1^), (**B**) time to reach *R*_N-max_ (*T*_RN-max_, h), (**C**) duration from 1/2 *R*_N-max_ to 1/2 *R*_N-max_ (*D*_1/2 max-RN_, h).

**Figure 6:**
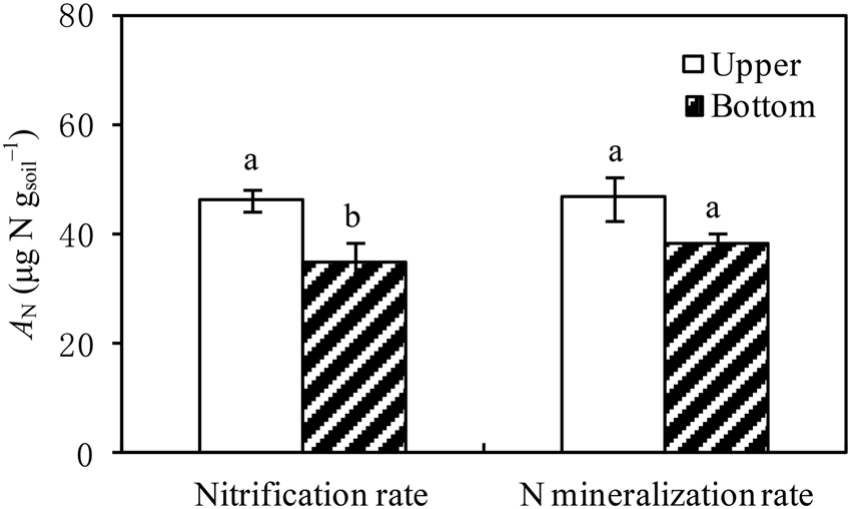
Accumulation of soil nitrogen mineralization and nitrification rates (*A*_N_, μg N g_soil_^−1^) during the 48-h incubation period.

Rewetting events also exerted strong pulse effects on *R*_N._ The observed *R*_N-max_ values were significantly higher in the upper location (40.87 µg N g_soil_^−1^ h^−1^) than that in bottom locations (22.86 µg N g_soil_^−1^ h^−1^), respectively (*P* < 0.05, Fig. 5A). *T*_N-max_ values were observed at 6 and 5 h in the upper and bottom locations, respectively. *D*_1/2max-RN_ values were estimated as 1.5 and 2.5 h for the upper and bottom samples, respectively. The values of *A*_N_ were calculated as 46.54 µg N g^−1^ (upper) and 38.25 µg N g^−1^ (bottom), which was significantly higher in the upper location (*P* < 0.001, Fig. 6). Similar to the *R*_C_ results, no significant relationships between *R*_N_ and MBN were observed from the start to peak *R*_N_ during the 48-h incubation period (data not shown).

### Relationships between *R*_C_ and *R*_N_ through drying-rewetting-drying

No correlation between *R*_*C*_ and *R*_N_ was observed through the drying-rewetting-drying processes. Even dividing the incubation processes as the maximum of MBN, *R*_C_ and *R*_N_ were not coupled in the early or late phase of the maximum of MBN in the total 48-h incubation period (all *Ps* > 0.05, Figs S2, S3, S4).

## Discussion

### Strong and rapid pulse effects of rewetting on *R*_C_

Rewetting exerted strong and rapid pulse effects on *R*_C_ in view of *R*_C-max_ and *T*_RC-max_. These pulse effects were observed immediately and peaked at about 10 min (Figs 1 and 2). The observed *R*_C-max_ values were about 80 to 90 times to the initial *R*_C_ values under air-drying conditions. Birch.H.F^5^ first reported that heavy precipitation may result in strong, short pulse effects on *R*_C_ and soil microbial growth in soils that have experienced long-term drought. Based on measurements of *R*_C_ after 15 and 30 min and 1, 2, 5, 24, and 48 h of simulated precipitation, Sponseller^10^ found that *R*_C_ responded immediately, peaked at 30 min, and returned to its original level within 48 h. Using a microorganism respiration apparatus at 25 °C with measurements at 3-h intervals, Griffiths^20^ found that *R*_C_ increased after 12 h with a peak at 24 h. Based on this research, we inferred that the shorter, stronger responses of *R*_C_ have not been detected by previous studies, resulting in underestimation of the pulse effects of rewetting (or precipitation events). A related study of Jones and Murphy^12^ indirectly supported the idea that the responses of soil microbes to simulated rainfall are significant in arable and grassland soil, with the greatest level of CO_2_ production occurring after about 16 min. Some studies have demonstrated that after the pulse effects, the CO_2_ release rate may return to its original level after 48 h, a longer period than that found in this study^10^. In this study, the optimal SWC (55% WHC) after simulated rewetting and temperature (22 °C), in part, should explain the observed stronger pulse effects. Furthermore, the pulse effects have been predicted to vary for different seasons, initial SWCs, and drying-wetting frequencies^1, 21, 22^, factors which require further investigation.

### Strong and short-term pulse effects of rewetting on *R*_N_

Rewetting exerted strong pulse effects on *R*_N_, which rapidly increased after simulated rewetting, with peak soil inorganic N content occurring at 5–6 h. This apparently delayed *R*_N_ (Figs 5 and 6). Precipitation events or drying-wetting processes are the main drivers of the N supply for plant growth in arid and semiarid areas^3, 23^. In this study, the content of soil inorganic N may reach the levels 35–45 µg N g_soil_^−1^ after 5–8 h of rewetting, suggesting a capacity for soil inorganic N supply from the short-term pulse effects of small precipitation events.

Pulse effects depend on the size, frequency, and duration of rewetting (or precipitation events)^1^. In arid and semiarid regions, most precipitation events are small^24–26^. For example, Sala and Lauenroth^26^ reported that precipitation events of 5 mm or less accounted for 70% of all precipitation events in a North American shortgrass steppe, while Zhang and Zhao^27^ found that these small events comprised 82% of precipitation events in the Heihe River basin. The pulse effects of small precipitation events may play important roles in the available supply of nutrients in arid and semi-arid regions, especially for some plant species with rapid growth and reproductive strategies^28, 29^. McIntyre, *et al*.^30^ concluded that riparian soils produced the greatest mineralization flush, as over 70% of the total mineralized N accumulated within 48 h of rewetting. Our findings provide direct evidence of short-term pulse effects of small precipitation events (rewetting) on *R*_N_, and we infer that increased precipitation quantity (or intensity) may trigger stronger and longer pulse effects, which should depend on the initial water levels of soils to a large extent^1^. An understanding of the dependence of pulse effects on precipitation intensity will be necessary for future explorations of biogeochemistry and the interactions between soil and plants in arid and semiarid regions. Furthermore, it is necessary to mention that our findings were concluded from one cycle of rewetting event, so our findings could be valid for further cycles, only if the pulse effect is maintained, which have been demonstrated in previous studies that dry-rewetting cycles regulate plant C rhizodeposition, stabilization and N cycling^29–31^. However, the substrate depletion and changes in soil microbial composition are inevitable in several cycles of rewetting events, resulting in a variable Birch effects (especially for intensity vs. duration). Therefore, experiments considering more cycles of rewetting events are required in future.

### Uncoupled pulse responses of *R*_C_ and *R*_N_ driven by soil microbes

Under microbial regulation, the pulse responses of *R*_C_ and *R*_N_ were asynchronous and uncorrelated. Our results support the assumption that the rapid growth of microbes and the microbial requirement of C:N stoichiometric stability^32, 33^ may result in uncoupled C and N mineralization through the short-term pulse effects of rewetting (or simulated precipitation). These findings differ from those of previous studies conducted under normal SWC conditions, which found that *R*_C_ and *R*_N_ were closely coupled in natural ecosystems^13^, and incubation experiments at weekly or monthly intervals^14, 15^.

**Figure 7:**
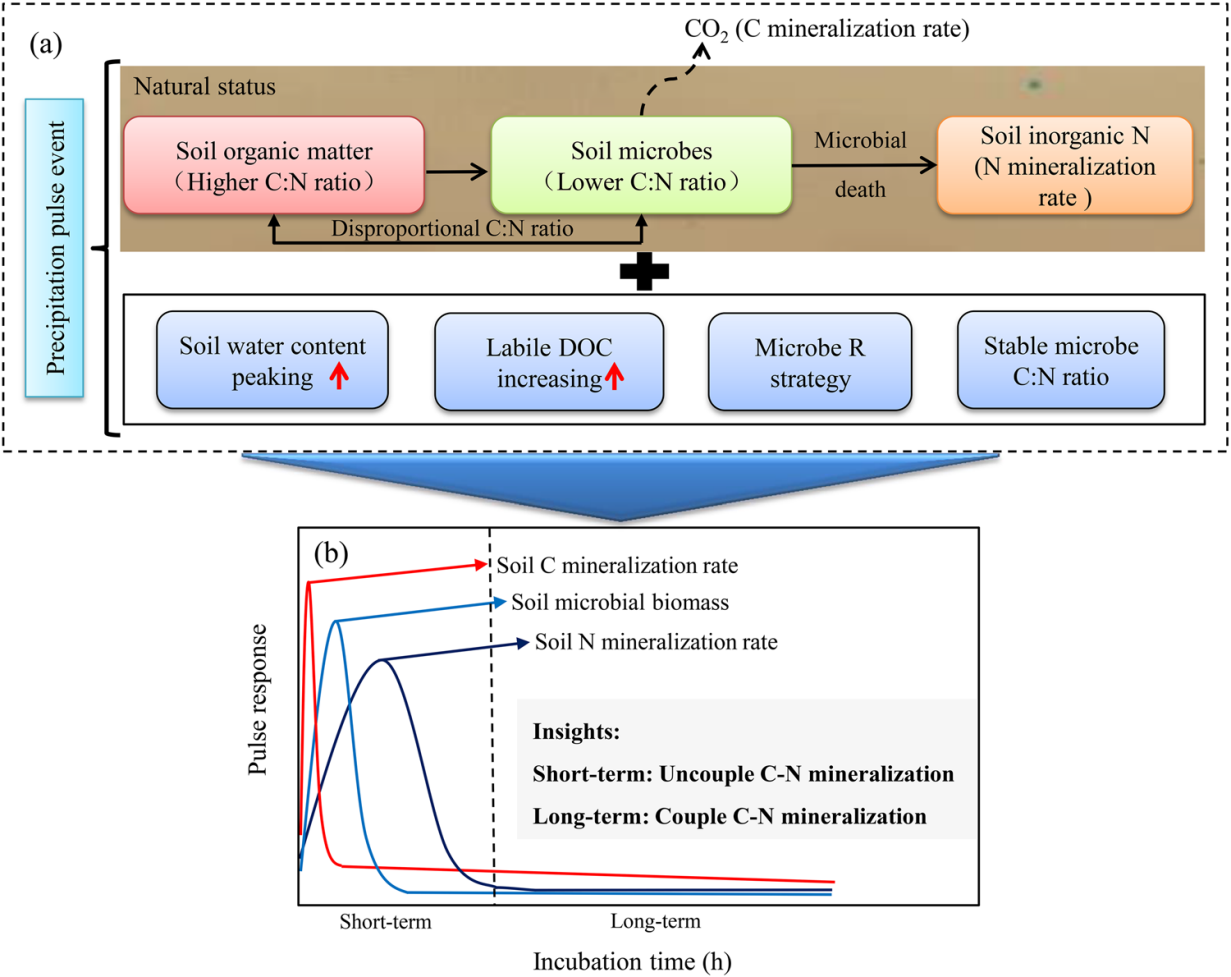
General concept of asynchronous pulse responses of soil carbon and nitrogen mineralization regulated by soil microbes to simulated rewetting.

Through the drying-rewetting process to stimulate a precipitation events (Fig. S1), soil microorganisms play important roles as reservoirs of soil nutrients and regulators of soil C and N mineralization rates^1, 16^, resulting in apparently asynchronous *R*_C_ and *R*_N_ processes and the observed uncoupled relationships. We developed a conceptual framework to explain the asynchronous pulse responses of *R*_C_ and *R*_N_ to simulated rewetting, regulated by soil microbes (Fig. 7). In detail, precipitation event rapidly enhance SWC and the availability of dissolved organic C, and therefore promote a sharp increase in soil microbial biomass (Fig. 2). Simultaneously, the higher C:N ratios of SOM and lower C:N requirements of soil microbes lead the microbes to decompose more SOM to satisfy their N requirement. This results in a sharp increase in CO_2_ emissions as surplus elements due to the relative stability of the C:N ratios in soil microbes. According to classical homeostasis theory in ecological stoichiometry, organisms have the capacity to maintain a stable C:N:P ratio despite differences in the availability of these elements^32, 34, 35^. Well-constrained C:N:P ratios of soil microbes have been reported recently using global meta-analysis^33^. With the rapid growth of soil microbes, the depletion of labile DOC and decreasing soil moisture result in an increasing microbial death and an apparent decrease in soil microbe biomass (supported by a sharp decrease in MBN shown in Fig. 2), and therefore, we can observed a rapid increase in soil N mineralization, accompanied by a steep decrease in soil C mineralization^36^. After several hours of precipitation, the C:N requirements of soil microbes and microbe biomass stabilize, and coupled soil C and N mineralization relationships may be observed^19^. Overall, the shifting roles of soil microbes from reservoirs to releaser for N may exemplify the key role of microbes in soil C and N mineralization during different phase, and which can explain the uncoupled relationships among *R*_N_, *R*_C_, and MBN at the initial phase of precipitation events. It is necessary to point out that, in this study, the data of MBC were not measured. Therefore, we assumed that MBN should be a proxy of MBC and microbial biomass some extent because of the stoichiometric homeostasis assumption of stable MBC:MBN ratio.

Rewetting events (or simulated precipitation) exert rapid, strong pulse effects on soil C and N mineralization rates over the short term. Microbes drive asynchronous processes in soil C and N mineralization rates and the uncoupled relationships among *R*_N_, *R*_C_, and MBN. This research provides a new conceptual framework of the pulse effects of rewetting (or small precipitation events) on soil C and N mineralization rates, as well as insights into the importance of these pulse effects in biogeochemistry and the interaction of plants and soils in arid and semiarid regions. It is necessary to mention that these findings were derived only from one incubation experiment, and therefore validation with more incubation experiments and field experiments are required in future.

## Materials and Methods

### Study area

Soil samples were collected from temperate forests northwest of Beijing (39° 24′N,116° 10′E). This region has a semi-arid temperate continental monsoon climate. The annual average temperature is approximately 11 °C, ranging from −1.4 °C in January to 22.4 °C in July. The annual average precipitation is about 626 mm. The regional soils are classified as Lixisols, according to the classification of world reference base for soil resources^37^. The dominant tree species are pine (*Pinus*) and maple (*Liquidambar formosana*). We established three sampling plots at two different elevations, designated as bottom (21.2 m.a.s.l.) and upper (500.1 m.a.s.l.). Selected soil properties of the two locations are provided in Table 1.

**Table 1:** Selected soil properties of the experimental plots.

Position	Location	Altitude (m.a.s.l.)	pH	Soil carbon content (%)	Soil nitrogen content (%)	C/N ratios
Upper	39.9901°N, 116.1717°E	500.1	8.14 ± 0.02^a^	2.26 ± 0.01^a^	0.19 ± 0.01^a^	12.06 ± 0.34^a^
Bottom	39.9667°N, 116.2086°E	21.2	7.56 ± 0.01^b^	3.40 ± 0.01^b^	0.20 ± 0.01^a^	17.16 ± 0.53^b^
F			5.57	0.07	0.23	1.59
*P*			<0.001	<0.001	0.30	<0.05

^†^Data are represented as means ± 1 SD (n = 3). Data with same superscript letters within each column indicated no significant difference at *P* = 0.05 level.

### Soil sampling and chemical analyses

Soil sampling was conducted in early November 2014. We randomly established three plots (10 × 10 m) at each location (bottom and upper) and collected 15 to 20 surface soil samples (0–10 cm) from each plot using a soil auger of 5 cm in diameter. The soil samples were sieved (< 2 mm), and roots and visible organic debris were removed by hand. Approximately 3 kg of each soil sample were air-dried for chemical analysis and incubation experiments.

The concentrations of soil C and N were measured using an elemental analyzer (vario MAX cube, ELEMENTAR, Germany). Soil pH was measured in a soil-water slurry (1:2.5, w/w) with an Ultrameter-2 pH meter (Myron L. Company, California, USA). Soil water holding capacity (WHC, %) and gravimetric water content (SWC, %) were measured using the interior-ring cutting and oven-drying weight.

### Incubation experiments and measurements of soil C and N mineralization rates

Soil samples were first air-dried at room temperature for approximately one week until SWC was less than 10% WHC. Ten-gram samples of air-dried soil were placed in incubation bottles (5 cm in diameter, 10 cm in height), with three replicates for each location. Basic soil respiration rates were measured at 22 °C (as detailed in section 4.3.1), and soil inorganic N content was measured (as detailed in section 4.3.2). Then, all soil samples were rewetted to 55% WHC (with approximately 2.5-3.1 mm of simulated precipitation) and incubated at 22 °C^38–40^. During the 48-h incubation period, *R*_C_ was continuously measured at 6-min intervals at 22 °C^41^. Furthermore, the SWC (%) of samples in the incubation bottles was measured at 0, 1, 2, 3, 4, 4.5, 5, 5.5, 6, 6.5, 7, 7.5, 8, 12, 24, and 48 h to determine the effects of drying-rewetting-drying (Fig. S1).

Similarly, ten-gram samples of air-dried soil were placed in incubation bottles (5 cm in diameter, 10 cm in height), with 57 replicates for each location, which were used for 19 separate destructive sampling times to measure the content of inorganic N (NO_3_^−^ and NH_4_^+^) and MBN.

#### Measurement of R_C_

A continuous-measurement apparatus developed by He, *et al*.^38^ and Dai, *et al*.^42^ was used to measure the *R*_C_ of each sample at 6-min intervals for 48 h. *R*_C_ was calculated using the slope of CO_2_ concentration and conversion factors^38, 43, 44^, as follows:

1 $${R}_{c}=\frac{c\times v\times \alpha \times \beta }{m}$$

where *R*_C_ is soil microbial respiration rate (μg C g_soil_^−1^ h^−1^), *C* is the slope of the change in CO_2_ concentration, *V* is the volume of the incubation bottle and gas tube, *m* is the soil weight (g), *α* is the conversion coefficient for CO_2_ mass, and *β* is a conversion coefficient of time. Here, *V* and *α* are used to transform volume concentration to mass concentration, and *β* is used to transform the measuring time from second to hour or day because the record of Licor 7000 is setup on per second basis.

#### Measurement of R_N_ and MBN

The contents of soil ammonium (NH_4_^+^-N) and nitrate (NO_3_^−^-N) were measured before and after incubation at intervals of 0, 1, 2, 3, 3.5, 4, 4.5, 5, 5.5, 6, 6.5, 7, 7.5, 8, 8.5, 9, 12, 24, and 48 h (3 replicates). In brief, 10-g soil samples were mixed with 25 mL of 0.5 M K_2_SO_4_ solution, shocked for 1 h, and then measured NH_4_^+^-N and NO_3_^−^-N with a filtered solution^45, 46^. NH_4_^+^-N, NO_3_^−^-N, and TN concentrations were determined with a flow analyzer (FUTURA, Alliance Instruments, France).

*R*_N_ (µg N g_soil_^−1^ h^−1^) was calculated using the following equations:

2 $${\rm{\Delta }}t={t}_{i+1}-{t}_{i}$$

3 $${R}_{amm}=L\times c{[{{\rm{NH}}}_{4}^{+}-{\rm{N}}]}_{i+1}-L\times c{[{{\rm{NH}}}_{4}^{+}-{\rm{N}}]}_{i}$$

4 $${R}_{{\rm{nit}}}=L\times c{[{{\rm{NO}}}_{3}^{-}-{\rm{N}}]}_{i+1}-L\times c{[{{\rm{NO}}}_{3}^{-}-{\rm{N}}]}_{i}$$

5 $${R}_{N}={R}_{amm}+{R}_{nit}$$

where *t*_i_ and *t*_i+1_ are the initial and ending times of incubation, respectively; Δ*t* is the duration of incubation; *c*[NH_4_^+^-N]_i_ and *c*[NH_4_^+^-N]_i+1_ are the concentrations of NH_4_^+^-N before and after incubation, respectively; *c*[NO_3_^−^-N]_i_ and *c*[NO_3_^−^-N]_i+1_ are the concentrations of NO_3_^−^-N before and after incubation, respectively; *L* is the volume of solution leached; and *R*_amm_, *R*_nit_, and *R*_N_ are the NH_4_^+^-N, NO_3_^−^-N, and total N mineralization rates on basis of per g dry soil, respectively.

Additionally, the contents of MBN were measured using the chloroform fumigation extraction method at intervals of 0, 1, 2, 3, 3.5, 4, 4.5, 5, 5.5, 6, 6.5, 7, 7.5, 8, 8.5, 9, 12, 24, and 48 h after rewetting, and each treatment has three replicates. In brief, the measurements were conducted using destructive sampling with the same treatments as in the measurements of *R*_N_. The soil samples were divided into two groups: 24-h fumigated (chloroform) and un-fumigated. Microbial biomass N (µgN g_soil_^−1^) was calculated as the difference in N extracted from the fumigated and un-fumigated samples under 1-h shake^47^.

### Characterizing the pulse effects of rewetting on R_*C*_ and R_*N*_

The following four parameters were selected to describe the pulse effects of rewetting on *R*_C_ and *R*_N_: (1) the maximum rates of *R*_C_ and *R*_N_ (*R*_C-max_ and *R*_N-max_, respectively); (2) the times to reach *R*_C-max_ and *R*_N-max_ (*T*_RC-max_ and *T*_RN-max_, respectively); (3) the duration of pulse effects from the start to the end of one-half *R*_C-max_ and *R*_N-max_ (*D*_1/2max-RC_ and *D*_1/2max-RN_, respectively); and (4) the accumulation of *R*_C_ and *R*_N_ during the 48-h incubation period (*A*_C_ and *A*_N_, respectively).

### Statistical analyses

Data were represented as mean and standard deviation (SD). *T*-tests were used to identify significant differences in soil properties between samples from the bottom and upper locations and the pulse effects (*R*_max_, *T*_R-max_, *D*_1/2Rmax_, and *A*). Regression analyses were used to evaluate the relationships between *R*_C_, *R*_N_, and MBN. Statistical analyses were performed using SPSS 13 (SPSS Inc., Chicago, IL, USA). The significance level was defined as *P* = 0.05.

## Electronic supplementary material

Supplementary information
