# Supplementary material for: Asynchronous pulse responses of soil carbon and nitrogen mineralization to rewetting events at a short-term: Regulation by microbes
Source: Sci Rep. 2017 Aug 8;7:7492. doi: 10.1038/s41598-017-07744-1 (PMC5548802; doi:10.1038/s41598-017-07744-1)
Supplement: Supplementary file 1 — Supplementary information [file 41598_2017_7744_MOESM1_ESM.pdf]

Supporting Information

Asynchronous pulse responses of soil carbon and nitrogen mineralization to rewetting events at a short-term: Regulation by microbes

Xiaoli Song [a, b, c], Jianxing Zhu [a,*], Nianpeng He [a], Jianhui Huang [b], Jing Tian [a], Xiang Zhao [c], Yuan Liu [a], Changhui Wang [b,*]

[a] Key Laboratory of Ecosystem Network Observation and Modeling, Institute of Geographic Sciences and Natural Resources Research, Chinese Academy of Sciences, Beijing 100101, China

[b] State Key Laboratory of Vegetation and Environmental Change, Institute of Botany, the Chinese Academy of Sciences, Beijing 100093, China

[c] College of Animal Science and Veterinary Medicine, Shanxi Agricultural University, Taigu 030801, China

*Correspondence and requests for materials should be addressed to J.Z (zhujianxing2008@yeah.net) and C.W (wangch@ibcas.ac.cn).

Tel.: +861068489040;

Fax: +861068489432.

Postal address: Institute of Geographic Sciences and Natural Resources Research,

Chinese Academy of Sciences. 11A, Datun Road, Chaoyang District, Beijing 100101, China

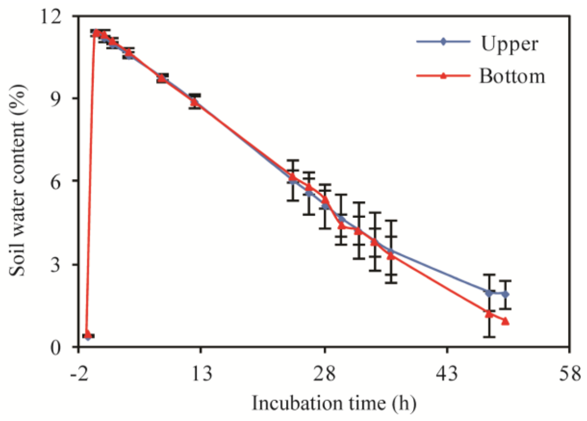

Fig. S1 Dynamics of soil water content (%) during the 48-h incubation period after rewetting

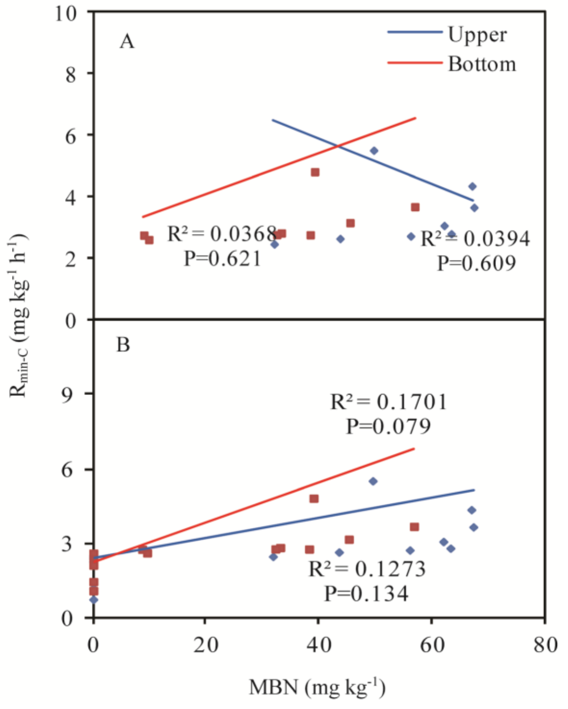

Fig. S2 Uncoupling relationships between soil carbon mineralization rate (R_{min-C}) and soil microbial biomass nitrogen (MBN). A, before the maximum carbon mineralization rate (max-R_{min-C}); B, total 48 h incubation.

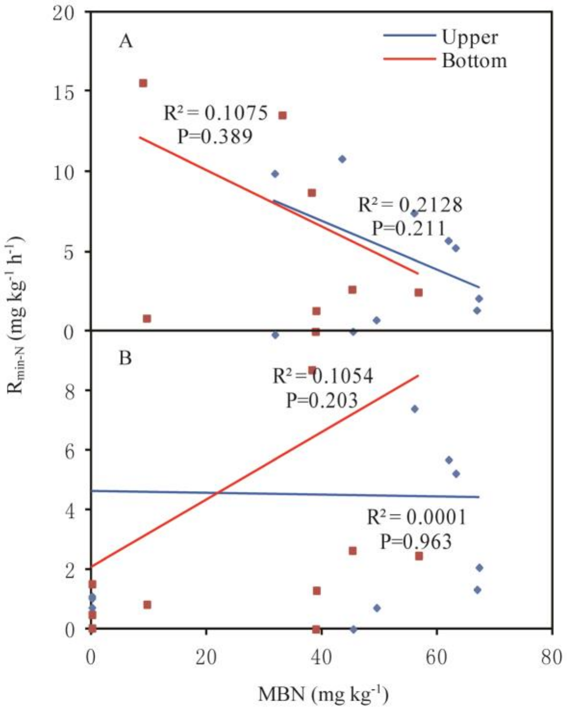

Fig. S3 Uncoupling relationships between soil nitrogen mineralization rate ($R_{min\text{-}N}$) and soil microbial biomass nitrogen (MBN). A, before the maximum nitrogen mineralization rate (max-$R_{min\text{-}N}$); B, total 48 h incubation.

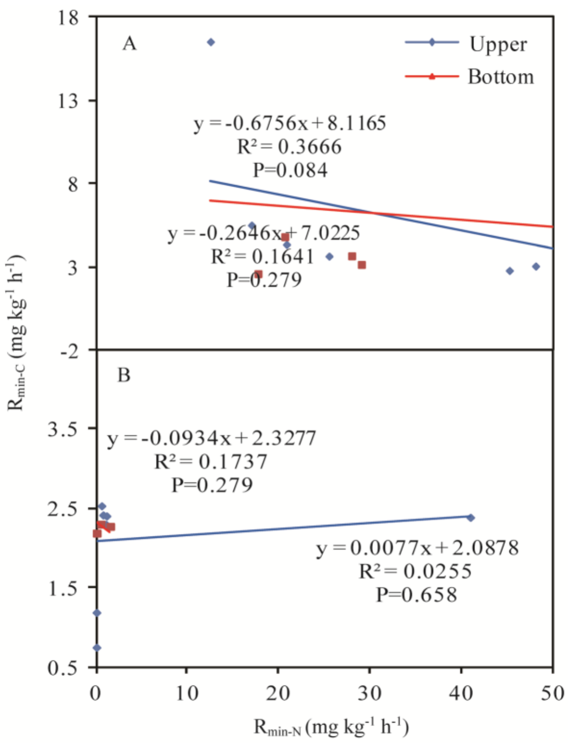

Fig.S4 The uncoupling relationships between soil carbon mineralization rate ($R_{min\text{-}C}$) and soil nitrogen mineralization rate ($R_{min\text{-}N}$). A, before the maximum nitrogen mineralization rate (6h); B, after 6 hour incubation time.
